# Design and Synthesis of a Novel Banana-Shaped Functional Molecule via Double Cross-Coupling

**DOI:** 10.3390/molecules24040698

**Published:** 2019-02-15

**Authors:** Bingchuan Yang, Guodong Shen, Xianqiang Huang, Rutao Liu

**Affiliations:** 1 School of Chemistry and Chemical Engineering, Liaocheng University, Liaocheng 252000, China; shenguodong@lcu.edu.cn (G.S.); hxq@lcu.edu.cn (X.H.); 2 School of Environmental Science and Engineering, Shandong University, Jinan 250100, China; 3 The Department of Chemistry, University of South Florida, 4202 East Fowler Avenue, Tampa, FL 33620, USA

**Keywords:** bent-core, Tröger’s base, double Sonogashira coupling

## Abstract

A novel banana-shaped molecule using 2,8-Dimethyl-6*H*,12*H*-5,11-methanodibenzo [*b,f*] [1,5]diazocine (Tröger’s base) as bent-core was synthesized via double Carbon-Carbon cross-coupling reaction. The double Sonogashira cross-coupling reaction was optimized by using Pd(PPh_3_)_2_Cl_2_ as catalyst, CuI as cocatalyst and diisopropylamine as base in place of triethylamine. The structure of this compound was confirmed by ^1^H-NMR, ^13^C-NMR, Fourier transform infrared (FT-IR) spectroscopy and mass spectrometry.

## 1. Introduction

Molecules bearing bent-cores have attracted extensive worldwide attention due to their outstanding properties. Some of these molecules have potential applications in asymmetric catalysis [1,2], and others have been wildly used in molecular recognition [3,4,5,6]. Banana-shaped molecules have rigid Λ shape, which will induce intermolecular polarity. Also because of their special structure, the molecules can pack closely and align in the direction of bending [7]. As a result, banana-shaped molecules with bent-cores and flexible tails can exhibit a variety of novel characteristics involving chirality, polarity, and liquid crystalline (LC) features [8,9,10,11,12]. Especially when bent-core is constructed in the liquid crystal molecules, this special bent shape promotes molecules packed in polar [3] and tilted [13] ways to form chiral lamellar phases such as the B2 and B7 [14,15,16].

Bent-core liquid crystal molecules also have nonlinear optical properties due to their unique shape and polar character, which have attracted considerable interest. What is more, polar characters of bent-shaped molecules were also used to develop ferroelectric switches or anti-ferroelectric materials [17].

This special usages have drawn great interest from not only scientific researchers but also industrial designers. Based on these fused properties, liquid crystals have been widely used in one-dimensional conductors, photoconductors, light emitting diodes, photovoltaic solar cells, and gas sensors [18,19,20,21].

**Scheme 1:**
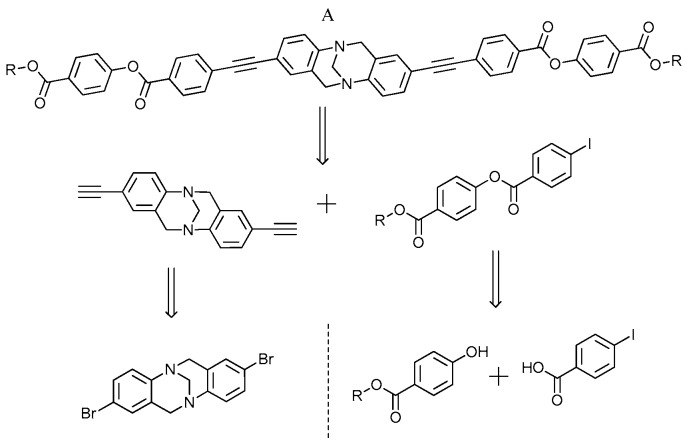
Retrosynthetic Analysis of target molecule A.

We have been focusing on the development of facile syntheses of heterocyclic systems [22,23,24,25,26] Herein, we choose bent-shaped 5,6,11,12-tetrahydrodibenzo[*b,f*] [1,5]diazocines (Tröger’s base like) as the bent-core to design and synthesize a novel banana-shaped molecule (Scheme 1). Tröger’s base was first synthesized by Tröger in 1887 [27], and its space structure is just similar to the letter of the Greek alphabet Λ. It has recently received renewed interest due to its inherent chirality and rigid C_2_ symmetry property [28,29,30,31,32,33,34,35,36,37,38,39]. Tröger’s base is a rigid and curved molecule due to its bridgehead stereogenic nitrogen atoms. A methylene strap connects the two chiral nitrogen atoms together, which is very suitable for the construction of liquid crystalline materials. To the different substituted groups, the dihedral angle between two benzene rings in Tröger’s base changes from 90° to 100°. For example, the dihedral angle between two aromatic rings in (5*S*,11*S*)-2,8- dibromo-6*H*,12*H*-5,11-ethanodibenzo[*b,f*] [1,5]diazocine is 94.5° [40], and the dihedral angle between two aromatic rings 1,2,3,4,7,8,9,10-octafluoro-6*H*,12*H*-5,11-methanodibenzo[*b,f*][1,5]-diazocine is 98.4° [41].

Tröger’s base has been constructed as a bent-core to bind different functional groups without ruining their original properties. And Tröger’s base can be used as molecular clefts because the bicyclic skeleton forces the molecule in a rigid locked conformation with the benzene rings in proximity. In supramolecular chemistry, Tröger’s base has been explored for the recognition of aliphatic dicarboxylic acids and enzyme inhibitors [42,43,44,45]. Enantiomerically enriched [46,47] analogues of Tröger’s base was also used in DNA intercalation and acted as ligands in asymmetric synthesis [48]. What is more, this Λ-shaped twisted configuration is theoretically disadvantageous for the formation of π–π close stacking, which may lead to fluorescence quenching in the solid state. But recent study revealed that the Λ-shaped Tröger’s base scaffolds can express aggregation-induced emission (AIE) properties [49,50].

## 2. Experimental Section

### 2.1. General

All chemicals purchased were used without purification. THF of analytical grade and de-ionized water were used throughout the experiment as solvents. ^1^H-NMR spectra were recorded on a Bruker Advance 400 (400 MHz) or 300 (300 MHz) spectrometer, using CDCl_3_ as solvent and tetramethylsilane (TMS) as internal standard. ^13^C-NMR spectra were recorded on a Bruker Advance 100 (100 MHz) or 75 (75 MHz) spectrometer, using CDCl_3_ as solvent and tetramethylsilane (TMS) as internal standard. HRMS spectra were determined on a Q-TOF6510 spectrograph (Agilent). Supplementary Materials: Representative experimental procedures, spectral data of compounds 3–8. This material is available free of charge via the internet.

### 2.2. Experimental Procedure for the Preparation of 2,8-Dibromo-6H,12H-5,11-methanodibenzo[b,f][1,5]diazocine (***2***)

**Figure 1:**
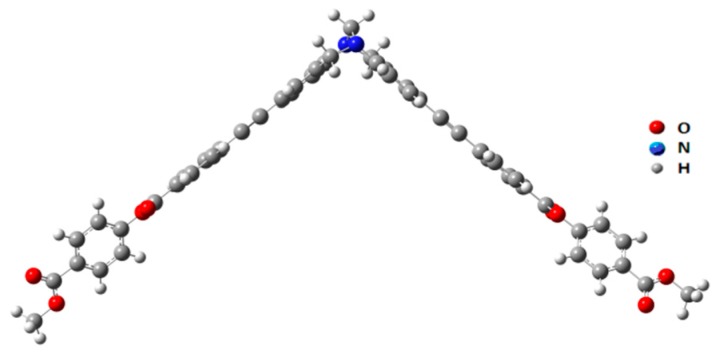
Ball-and-Stick model of compound **8**.

To a stirred mixture of the 4-bromoaniline (**1**) (3.44 g, 30 mmol), paraformaldehyde (5.40 g, 60 mmol) was added with TFA (100 mL; 1.3 mol), the mixture was stirred for 168 h at room temperature Then, TFA was removed in vacuo, water (100 mL) was added followed by the addition of saturated aqueous NH_3_ solution (100 mL). The aqueous layer was extracted with dichloromethane (3 × 50 mL). The combined organic layers were dried (MgSO_4_), filtered, concentrated in vacuo and purified by flash chromatography on silica gel to gain the product **2** (PE:EtOAc = 20:1, 3.363 g, 88%). As shown in Figure 1. ^1^H-NMR (DMSO, TMS, 400 MHz): 4.08 (d, *J* = 16.8 Hz, 1H), 4.2 (s, 1H), 4.62 (d, *J* = 16.8 Hz, 1H), 6.87 (d, *J* = 8.48 Hz, 1H), 7.23 (d, *J* = 8.08 Hz, 1H), 7.46 (d, *J* = 8.56 Hz, 1H). ^13^C-NMR (CDCl_3_, TMS, 100 MHz): 58.3, 66.7, 116.8, 126.7, 129.7, 129.7, 130.6, 146.8.

### 2.3. Experimental Procedure for the Preparation of 2,8-Bis((trimethylsilyl)ethynyl)-6,12-dihydro-5,11-methanodiben-zo[b,f][1,5]diazocine (***3***)

**Figure 2:**
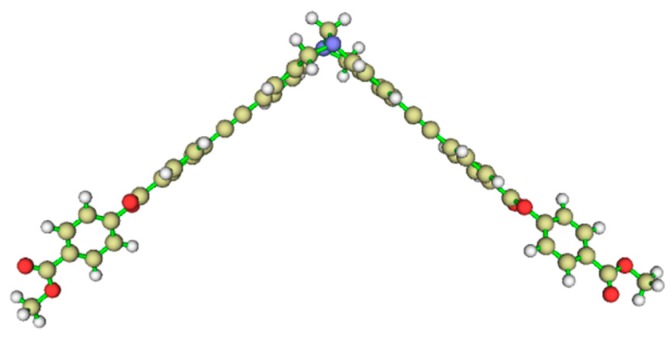
The optimized structure of **8** calculated by Gaussian 09 program using the B3LYP method with the 6-31G + (d,p) basis set [51].

A Schlenk tube containing 2,8-dibromo-6,12-dihydro-5,11-methanodibenzo [*b,f*][1,5] diazocine (**2**) (0.38 g, 1 mmol), dichlorobis(triphenylphosphine)palladium(II) (0.07 g, 0.1 mmol), copper(I) iodide (0.012 g, 0.06 mmol), triphenylphosphine (0.052 g, 0.2 mmol) was degassed on a vacuum line by repeatedly alternating between an argon atmosphere and vacuum. Then diisopropylamine (30 mL) was added dropwisely. Finally, a solution of trimethylsilylacetylene (0.53 mL, 3 mmol) was added dropwise and the mixture was stirred at 50 °C for 11 h. After that the mixture was filtered through a pad of celite and dichloromethane (30 mL) was used to wash the additional product through the celite pad. The filtrate was evaporated to dryness and purified by flash chromatography on silica gel to gain the product **3** (PE:EtOAc = 6:1, 0.250 g, 60%).As shown in Figure 2. ^1^H-NMR (CDCl_3_, 300 MHz) δ 0.20 (s, 18H), 4.10 (d, *J* =16.8 Hz, 2H), 4.27 (s, 2H), 4.62 (d, *J* =16.8 Hz, 2H), 7.01 (d, *J* = 8.4 Hz, 4H), 7.24 (dd, *J* = 8.7, 2.4 Hz, 2H). ^13^C-NMR (CDCl_3_, TMS, 100 MHz): 0.00, 58.5, 66.8, 93.4, 104.8, 118.6, 124.8, 127.7, 130.7, 131.0, 148.3.

### 2.4. Experimental Procedure for the Preparation of 2,8-Diethynyl-6,12-dihydro-5,11-methanodibenzo[b,f][1,5]diazoci-ne (***4***)

**Figure 3:**
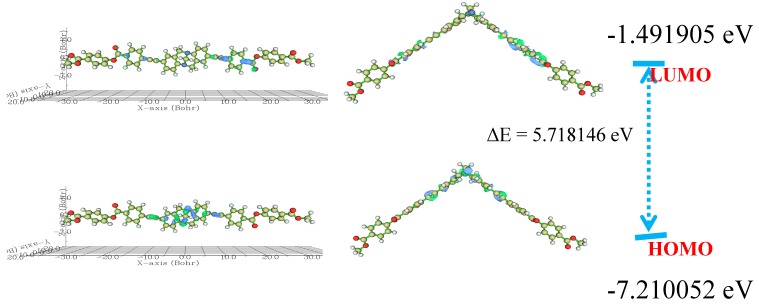
HOMO/LUMO imagines of **8** calculated by Gaussian 09 program using the B3LYP method with the 6-31G + (d,p) basis set [51].

2,8-bis((trimethylsilyl)ethynyl)-6,12-dihydro-5,11-methanodi- benzo[*b,f*][1,5] diazocine (0.150 g, 0.362 mmol) was dissolved in THF/MeOH (V_THF_:V_MeOH_ = 1:1) 50 mL and potassium carbonate was added (0.333 g, 2.461 mmol). The mixture was stirred for 5 h, filtered, concentrated in vacuo and purified by flash chromatography on silica gel to gain the product 4 (PE:EtOAc = 6:1, 0.092 g, 95%). As shown in Figure 3. ^1^H-NMR (CDCl_3_, 300 MHz) δ 2.97 (s, 2H), 4.13 (d, *J* = 16.8 Hz, 2H), 4.29 (s, 2H), 4.65 (d, *J* = 16.8 Hz, 2H), 7.07 (d, *J* = 8.1 Hz, 4H), 7.24 (dd, *J* = 8.1 Hz, 1.5 Hz, 2H); ^13^C-NMR (CDCl_3_, 300 MHz) δ 58.4, 66.7, 76.5, 76.6, 77.0, 77.5, 83.3, 117.6, 125.0, 127.8, 130.9, 131.2, 148.5.

### 2.5. Experimental Procedure for the Preparation of 4-(Methoxycarbonyl)phenyl-4-iodobenzoate (***7***)

One equivalent of p-Iodobenzoic acid (**5**) (0.250 g, 1 mmol), methyl 4-hydroxybenzoate (**6**) (0.152 g, 1 mmol), DCC (0.206 g, 1 mmol) and DMAP (0.025 g, 0.2 mmol) were dissolved in dimethyl carbonate (DMC) 30 mL and stirred at room temperature for 10 h. The whole mixture was filtered and the filtrate was distilled by vacuum. The crude product was purified by flash chromatography on silica gel to gain the product 7 (PE:EtOAc = 6:1, 0.308 g, 80%). ^1^H-NMR (CDCl_3_, 400 MHz) δ 3.93 (s, 6H), 4.73 (d, *J* = 16.8 Hz, 2H), 7.29 (d, *J* = 8.7 Hz, 2H), 7.89 (s, 4H), 8.12 (d, *J* = 8.7 Hz, 2H); ^13^C-NMR (CDCl_3_, 400 MHz) δ 52.2, 102.0, 121.7, 128.0, 128.6, 131.3, 131.6, 138.1, 154.4, 164.2, 166.3.

### 2.6. Experimental Procedure for the Preparation of Bis(4-(methoxycarbonyl)phenyl)4,4′-((6,12-dihydro-5,11-methano-dibenzo[b,f][1,5]diazocine-2,8-diyl)bis(ethyne-2,1-diyl))diben-zoate (***8***)

A Schlenk tube containing 2,8-diethynyl-6,12-dihydro-5,11-methanodibenzo[*b,f*][1,5]diazocine (97 mg, 0.36 mmol), 4-(methoxycarbonyl)phenyl 4-iodobenzoate (276 mg, 0.72 mmol), dichlorobis(triphenylphosphine)palladium(II) (25 mg, 0.036 mmol), copper(I) iodide (3 mg, 0.018 mmol), triphenylphosphine (19 mg, 0.072 mmol) and dry triethylamine (20 mL) was degassed on a vacuum line by repeatedly alternating between an argon atmosphere and vacuum. The reaction mixture was stirred at 50 °C for 18 h. The reaction mixture was then filtered and evaporated to dryness. The product was purified by column chromatography on silica gel using CHCl_3_:EtOAc = 15:1 as the eluent to gain the product 8 (141 mg, 50%). white solid; m.p. 210 °C. IR (KBr), ν_max_ (cm^−1^): 2946, 2900, 2202, 1733, 1713, 1600, 1506. ^1^H-NMR (CDCl_3_, 400 MHz) δ 3.93 (s, 6H), 4.22 (d, *J* = 16.8 Hz, 2H), 4.35 (s, 2H), 4.73 (d, *J* = 16.8 Hz, 2H), 7.15 (d, *J* = 9.8 Hz, 4H), 7.30 (d, *J* = 8.1 Hz, 2H), 7.39 (dd, *J* = 8.3, 1.7 Hz, 2H), 7.60 (d, *J* = 8.5 Hz, 4H), 8.14 (t, *J* = 9.8 Hz, 14.1 Hz, 8H); ^13^C-NMR (CDCl_3_, 400 MHz) δ 52.2, 58.5, 66.8, 88.0, 92.9, 118.0, 121.7, 125.2, 127.9, 128.0, 128.2, 129.1, 130.2, 130.6, 131.0, 131.3, 131.6, 148.8, 154.5, 164.1, 166.3; HRMS (EI): Calcd for C_49_H_34_N_2_O_8_, 779.2388; found, 779.2383.

### 2.7. Theoretical Study

Computational methods. All calculations were conducted using the B3LYP functional (Gaussian 09, Revision B. 01.; Gaussian, Inc.: Wallingford, 2009) combined with the 6-31+(d,p) basis set, as implemented in Gaussian 09 software package (Gaussian 09, Revision B. 01.; Gaussian, Inc.: Wallingford, 2009). Vibrational analyses at the same level of theory were also carried out to confirm all stationary points as minima (zero imaginary frequencies) or first-order saddle points (one imaginary frequency) and to provide free energies at 298.15 K.

## 3. Results and Discussion

**Scheme 2:**
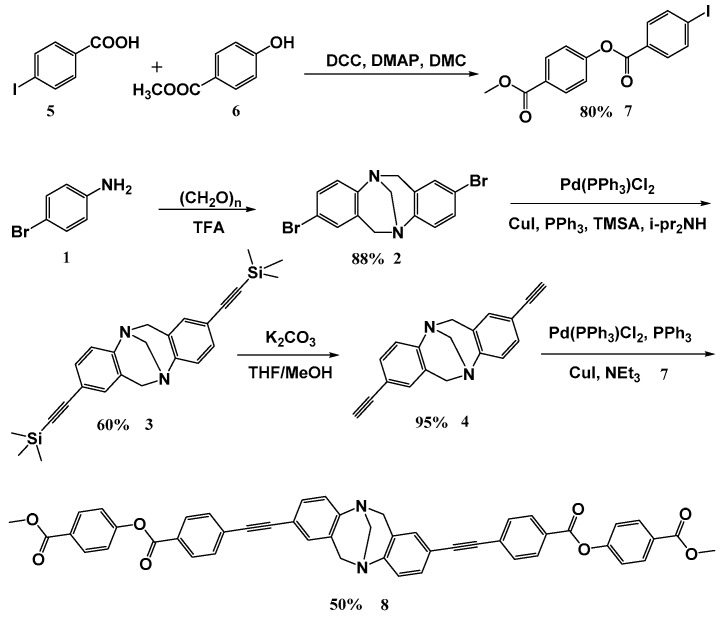
Synthesis of Tröger’s base like derivative.

As shown in Scheme 2, starting from 4-bromoaniline **1**, rac-(±)-Tröger’s base **2** was synthesized by condensation of **1** with paraformaldehyde in trifluoroacetic acid (TFA) according to the procedure described in Scheme 1. Tröger’s base derivative **2** could react with trimethylsilylacetylene (TMSA) smoothly to get high yield product **3** under the common conditions of Sonogashira cross-coupling reaction by using Pd(PPh_3_)_2_Cl_2_ as catalyst, CuI as cocatalyst and diisopropylamine as base in place of triethylamine. Then the trimethylsilyl groups of **3** was removed to obtain the product **4** in excellent yield under the conditions of K_2_CO_3_ as the base and V_THF_:V_MeOH_ = 1:1 as solvent. Intermediate **7** was obtained from p-iodobenzoic acid and Methyl 4-hydroxybenzoate under the conditions of *N*,*N*′-dicyclohexylcarbodiimide (DCC) as dehydrant and 4-dimethylaminopyridine (DMAP) as catalyst in the solvent of dimethyl carbonate (DMC). At the end, the intermediate **7** could react with **3**, to obtain target molecule **8** [14] in high yield under the conditions of PdCl_2_(PPh_3_)_2_ and CuI as cocatalyst and triethylamine as base.

We slightly modified the Sonogashira cross-coupling reaction conditions by using diisopropylamine as base instead of triethylamine, and the reaction temperature changed from room temperature to 50 °C improving the yield of 2,8-dibromo-6,12-dihydro-5,11-methano-dibenzo[*b,f*][1,5]diazocine **6** to 60% successfully. Finally, the target molecule was obtained in 26% overall yield in five steps.

To understand the structure changes of **8**. We carried out density functional theory (DFT) calculation by Gaussian 09 program using the B3LYP method with the 6-31G + (d,p) basis set. The optimized structure shown in Figure 1 and Figure 2 has a clear bent-core configuration, however the tails of the molecule are not flexible enough. The HOMO-LUMO energy gap of compound **8** is 5.718146 eV (Figure 3). Electron cloud transferred from the Tröger’s base like core to the benzene rings when the molecule excitated from HOMO to LUMO. According to the research results, this molecule has potential application in liquid crystal field and further study will be conducted.

## 4. Conclusions

In summary, we have designed and synthesized a novel banana-shaped molecule based on Tröger’s base via double Sonogashira cross-coupling reaction. The chemical structures have been characterized by ^1^H-NMR, ^13^C-NMR, Fourier transform infrared (FT-IR) spectroscopy and mass spectrometry. Theoretical study was also conducted by Gaussian 09 program, and we confirmed that the molecule has a bent-core structure. In the future, we will use this method to obtain a series of these bent-core compounds. Further studies on its applications in the photology are currently in progress.

## Supplementary Materials

Representative experimental procedures, spectral data of compounds **3**–**8**. This material is available free of charge via the internet.

## Date Availability

The data used to support the findings of this study are available from the corresponding author upon request.
