# Supplementary material for: Design and Synthesis of a Novel Banana-Shaped Functional Molecule via Double Cross-Coupling
Source: Molecules. 2019 Feb 15;24(4):698. doi: 10.3390/molecules24040698 (PMC6412220; doi:10.3390/molecules24040698)
Supplement: Supplementary file 1 [file molecules-24-00698-s001.pdf]

Supporting Information

Design and Synthesis of a Novel Banana-Shaped Functional Molecular *via* double cross-coupling

Bingchuan Yang,[a,b,c]* Guodong Shen,[a] Xianqiang Huang,[a] Rutao Liu[b]*

[a] *School of Chemistry and Chemical Engineering, Liaocheng University, Liaocheng, 252000, P R China.*
[b] *School of Environmental Science and Engineering, Shandong University, Jinan, 250100, P R China.*
[C] *The Department of Chemistry, University of South Florida, 4202 East Fowler Avenue, Tampa, Florida 33620, United States*

Measurements

The FT-IR spectra were recorded on a VERTEX-70 plus spectrometer. ^{1}H, ^{13}C NMR spectra were recorded on a Bruker Avance 400 spectrometer (9.4 T, 400.1 MHz for ^{1}H NMR and 100.6 MHz for ^{13}C NMR) on samples in $CDCl_3$ at room temperature using tetramethylsilane (TMS) as an internal standard. Mass spectra were obtained from Agilent Q-TOF6510.

Materials

Triethylamine was dealed with sodium and diphenyl ketone before use. Unless otherwise noted, all the other chemical were commercially available and use as received. 4-bromoaniline, $Pd(PPh_3)_2Cl_2$, bis(trimethylsilyl)acetylene (TMSA) (J&K Chemical), N, N'-dicyclohexylcarbodiimide (DCC) and N, N'-dimethylpyridin-4-amine (DMAP) (aladdin) were purchased, respectively.

Synthesis

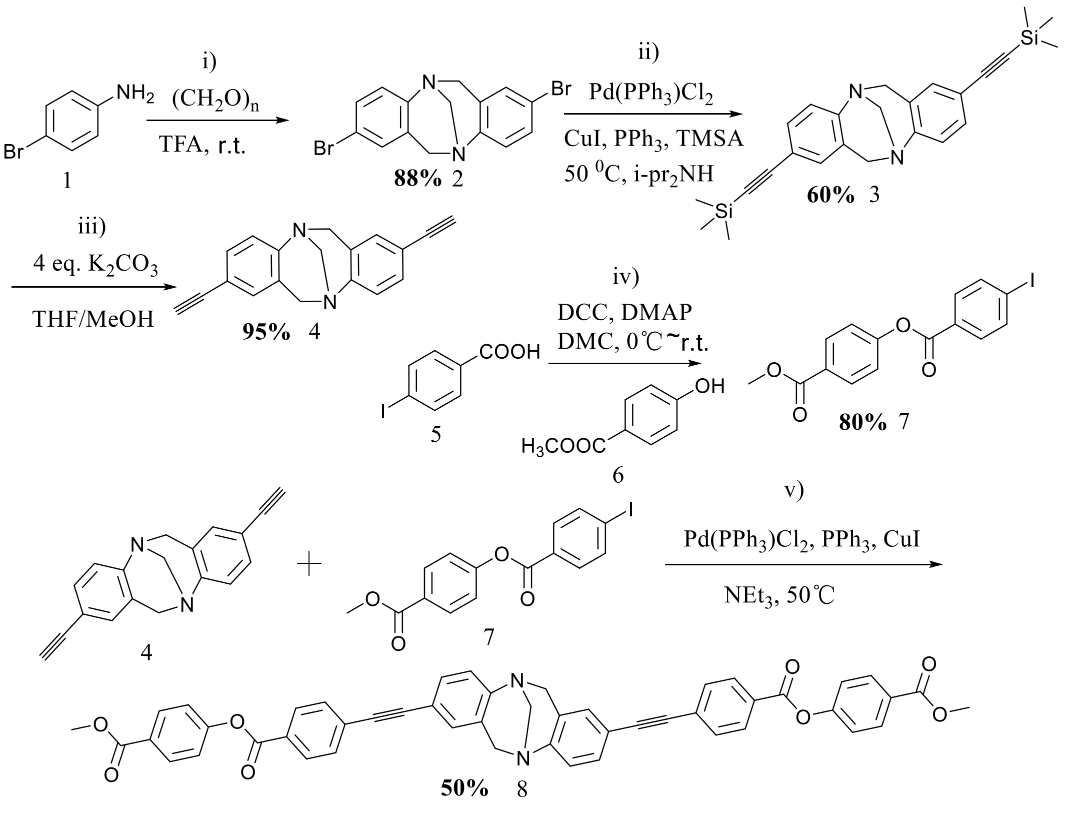

Scheme 1. Synthesis of target molecule 8

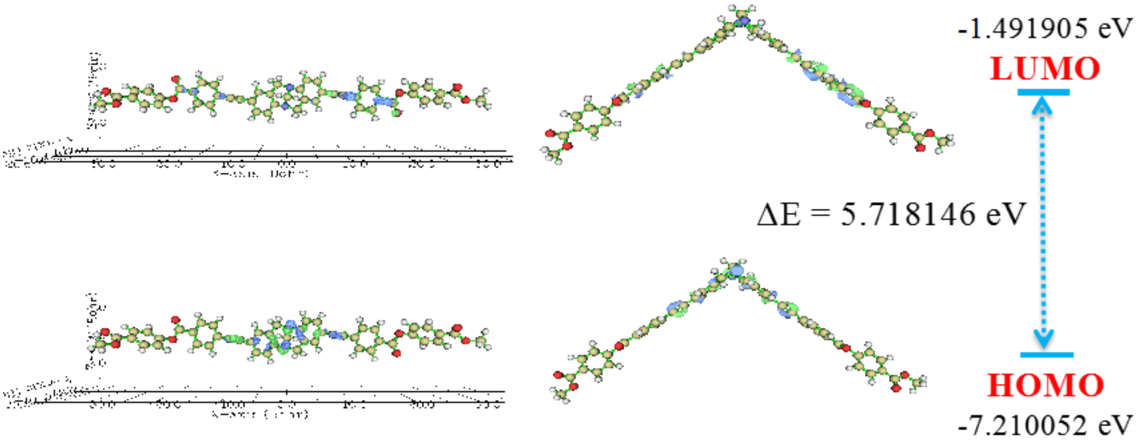

Scheme 1. HOMO/LUMO imagines of **8** calculated by Gaussian 09 program using the B3LYP method with the 6-31G + (d,p) basis set.

Cartesian Coordinates and Absolute Energies for optimized structure

	X	Y	Z
C	-2.79628271	4.51577567	0.51329105
C	-1.72764087	5.38934431	0.32547616
C	-1.30508540	5.68413345	-0.98094434
C	-1.93922892	5.07233328	-2.06921206
C	-2.99058735	4.19313128	-1.87364590
C	-3.43953494	3.91023727	-0.57174428
H	-3.12882332	4.28169396	1.52190025
H	-1.60729846	5.32327177	-3.07260214
H	-3.48534207	3.72851750	-2.71997162
N	-0.24445009	6.60779948	-1.22914023
C	0.01272976	7.44073050	-0.05903112
H	0.88576886	8.06619859	-0.26295263
C	-1.01280367	6.00351287	1.51815084
H	-0.81834574	5.23584239	2.27236691
C	1.01654364	5.95667778	-1.60672702
H	1.65634213	6.70556931	-2.08945124
H	0.81010571	5.17755788	-2.34591928
N	0.25785183	6.62768490	1.12761938
C	1.72201742	5.35567154	-0.40201326
C	1.30419677	5.68344266	0.89811010
C	2.77749844	4.46249903	-0.57181721
H	3.10624898	4.20272903	-1.57533723
C	1.92897659	5.08429494	1.99868449
H	1.60089762	5.36066253	2.99661747

C	3.41169279	3.86979713	0.52546053
C	2.96697681	4.18570738	1.82125872
H	3.45465479	3.73095702	2.67700896
C	4.49306856	2.95346226	0.32958860
C	5.41158759	2.17932753	0.16082225
H	-1.64090686	6.77148316	1.98596063
C	-4.53352224	3.01369997	-0.35542842
C	-5.46138084	2.25575344	-0.16551978
C	-6.55457584	1.36435399	0.07127614
C	-6.91797804	1.02693721	1.38621543
C	-7.27535933	0.82046190	-1.00410247
C	-7.97980479	0.16579481	1.61618883
H	-6.35993587	1.44872174	2.21526895
C	-8.33774330	-0.04252747	-0.77096337
H	-6.99351688	1.08331713	-2.01802926
C	-8.69242864	-0.37183296	0.54059625
H	-8.27370661	-0.10413246	2.62530202
H	-8.89509866	-0.46255152	-1.60079592
C	-9.82205077	-1.28587060	0.85738255
O	-10.17673203	-1.57877314	1.96983659
O	-10.41322928	-1.76633865	-0.27312792
C	-11.50470832	-2.61536978	-0.16177494
C	-12.62757429	-2.26672179	0.58470472
C	-11.45237184	-3.79571797	-0.89454263
C	-13.71377346	-3.13158033	0.59672902
H	-12.63999291	-1.34444526	1.15248053
C	-12.54586969	-4.65470513	-0.87837183
H	-10.55942307	-4.02552785	-1.46550087
C	-13.67782131	-4.32425690	-0.13029759
H	-14.60482100	-2.89587431	1.16927451
H	-12.52497655	-5.58022579	-1.44242336
C	-14.87476834	-5.20893021	-0.07356297
O	-15.88057215	-4.95670442	0.54993643
O	-14.72263321	-6.33277109	-0.79393704
C	-15.83820331	-7.22543521	-0.76774891
H	-15.54514860	-8.08067809	-1.37374798
H	-16.05252993	-7.53138010	0.25832517
H	-16.72144459	-6.73810604	-1.18617384
H	-0.85082952	8.08324725	0.13196991

C	6.49602243	1.27078500	-0.04949110
C	7.19982187	0.73806895	1.04260884
C	6.86928289	0.90675030	-1.35447761
C	8.25654751	-0.13840908	0.83557371
H	6.90976035	1.02087634	2.04878281
C	7.92514263	0.03190004	-1.55839104
H	6.32392245	1.31923241	-2.19656520
C	8.62209188	-0.49308776	-0.46633532
H	8.80126844	-0.54870554	1.67856818
H	8.22688977	-0.25820413	-2.55953139
C	9.74876512	-1.41954882	-0.75613235
O	10.10999035	-1.73573666	-1.86023622
O	10.33022328	-1.87944657	0.38767949
C	11.42374039	-2.72878748	0.30210774
C	12.54653799	-2.39789988	-0.45173049
C	11.37348739	-3.88934266	1.06704743
C	13.63720124	-3.25896134	-0.44210464
H	12.55658336	-1.49115097	-1.04397503
C	12.47027540	-4.74225651	1.07367526
H	10.48060693	-4.10715434	1.64280168
C	13.60220929	-4.43144433	0.31806597
H	14.52047120	-3.02399489	-1.02511970
H	12.46561377	-5.65613479	1.65844326
C	14.74601628	-5.38498510	0.35602668
O	14.76029338	-6.40689797	1.00421744
O	15.77424959	-4.99161815	-0.41312429
C	16.89677932	-5.87602291	-0.42500628
H	16.59698088	-6.86044865	-0.79025486
H	17.62320744	-5.41992969	-1.09502491
H	17.30761549	-5.97867287	0.58144878

Figures

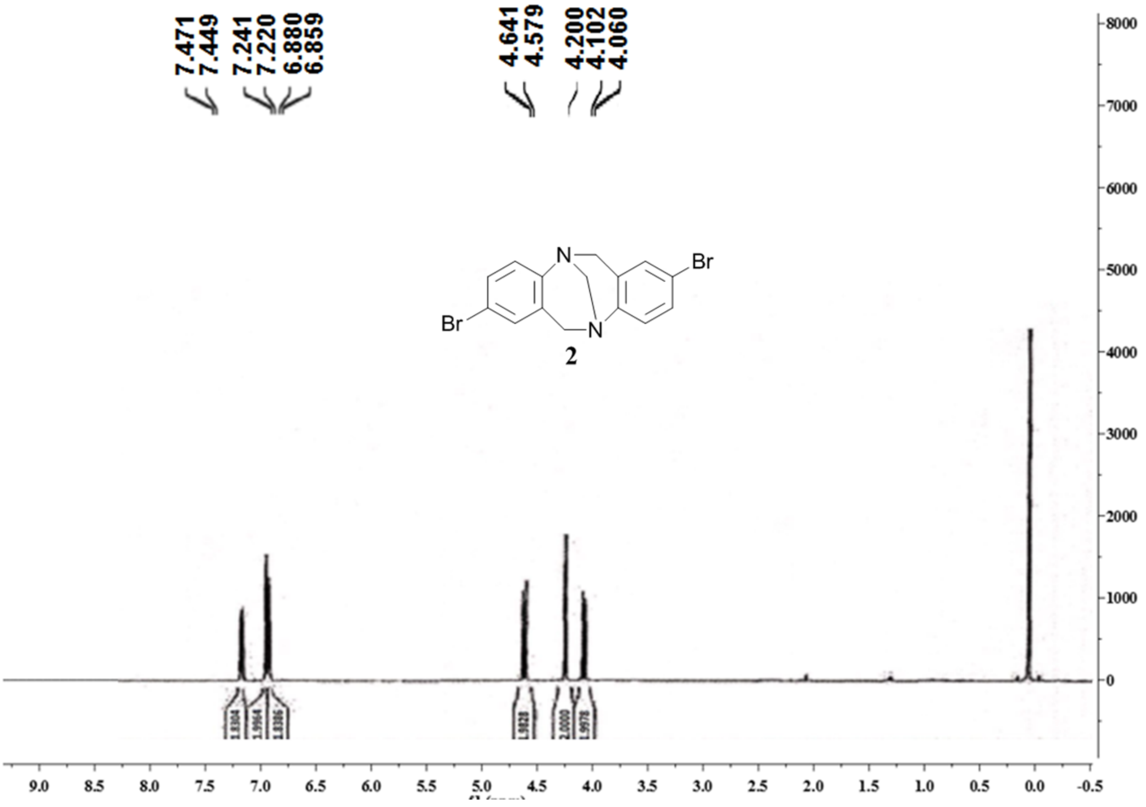

Figure 1(a). ^{1}H NMR spectrum of 2 (400MHz, $CDCl_3$)

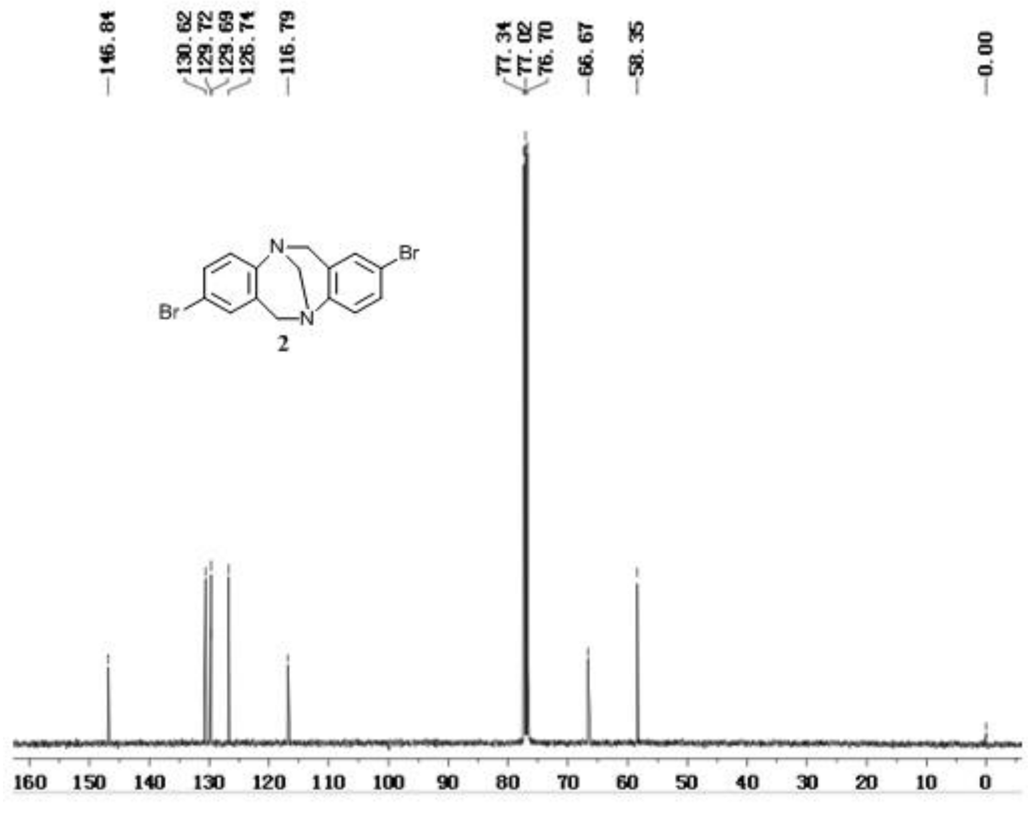

Figure 1(b). ^{13}C NMR spectrum of **2** (100MHz, $CDCl_3$).

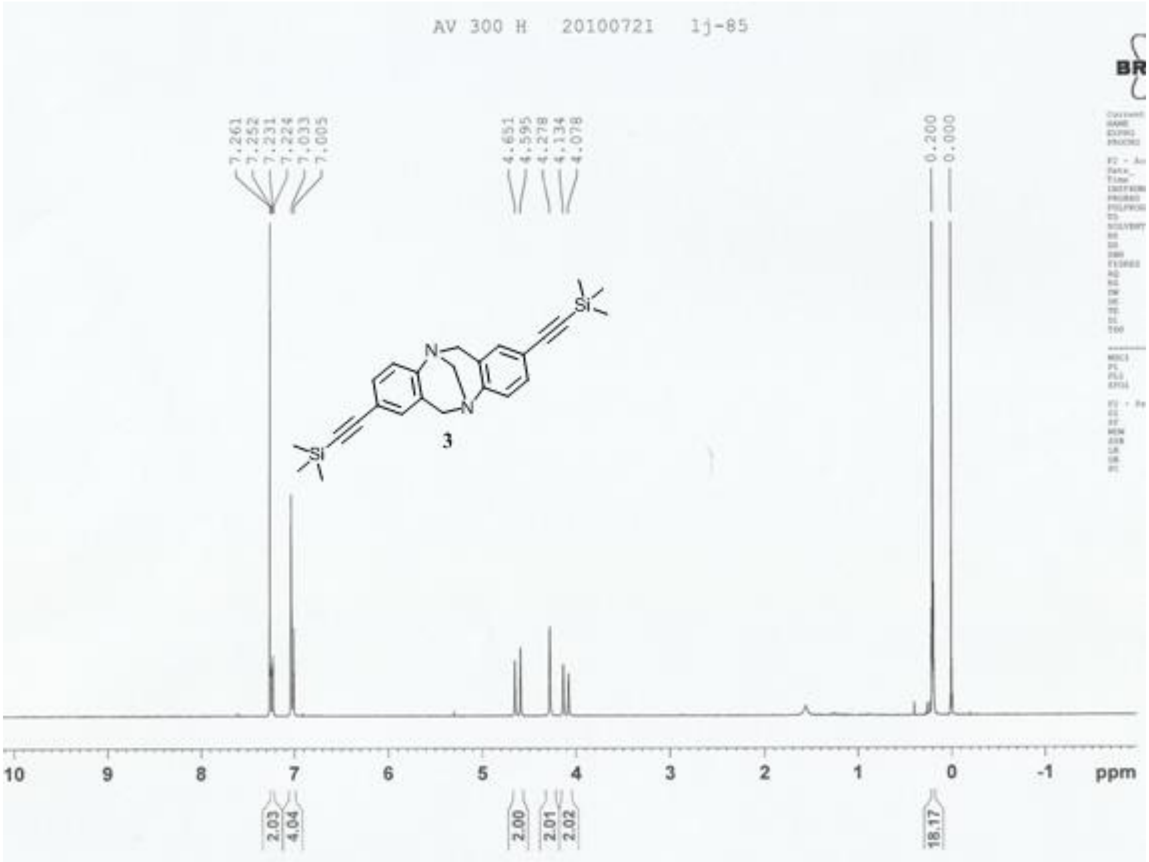

Figure 2(a). ^{1}H NMR spectrum of **3** (400MHz, $CDCl_3$)

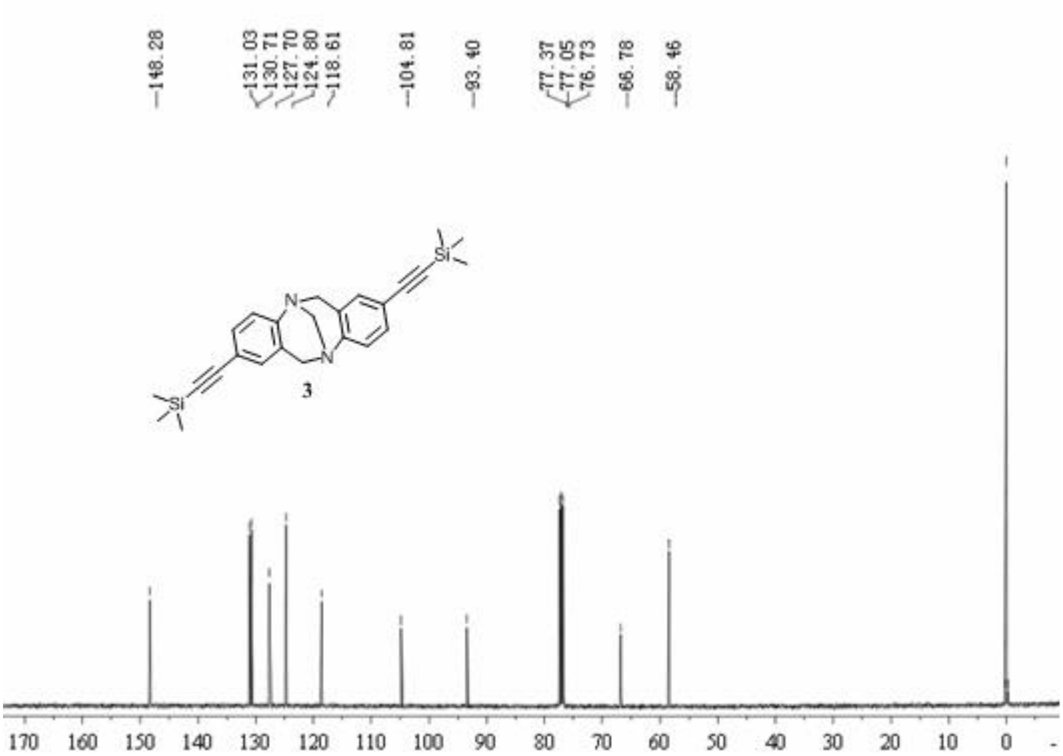

Figure 2(b). ^{13}C NMR spectrum of **3** (100MHz, $CDCl_3$)

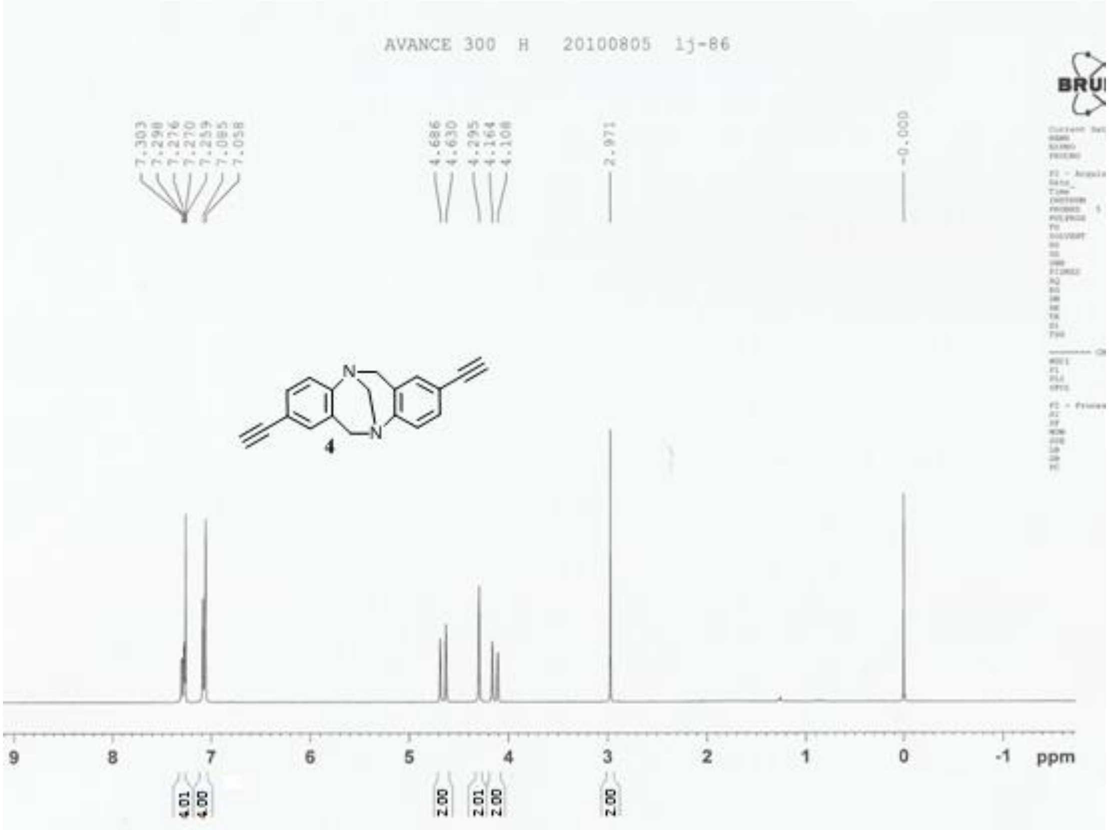

Figure 3(a). ^{1}H NMR spectrum of **4** (400MHz, $CDCl_3$)

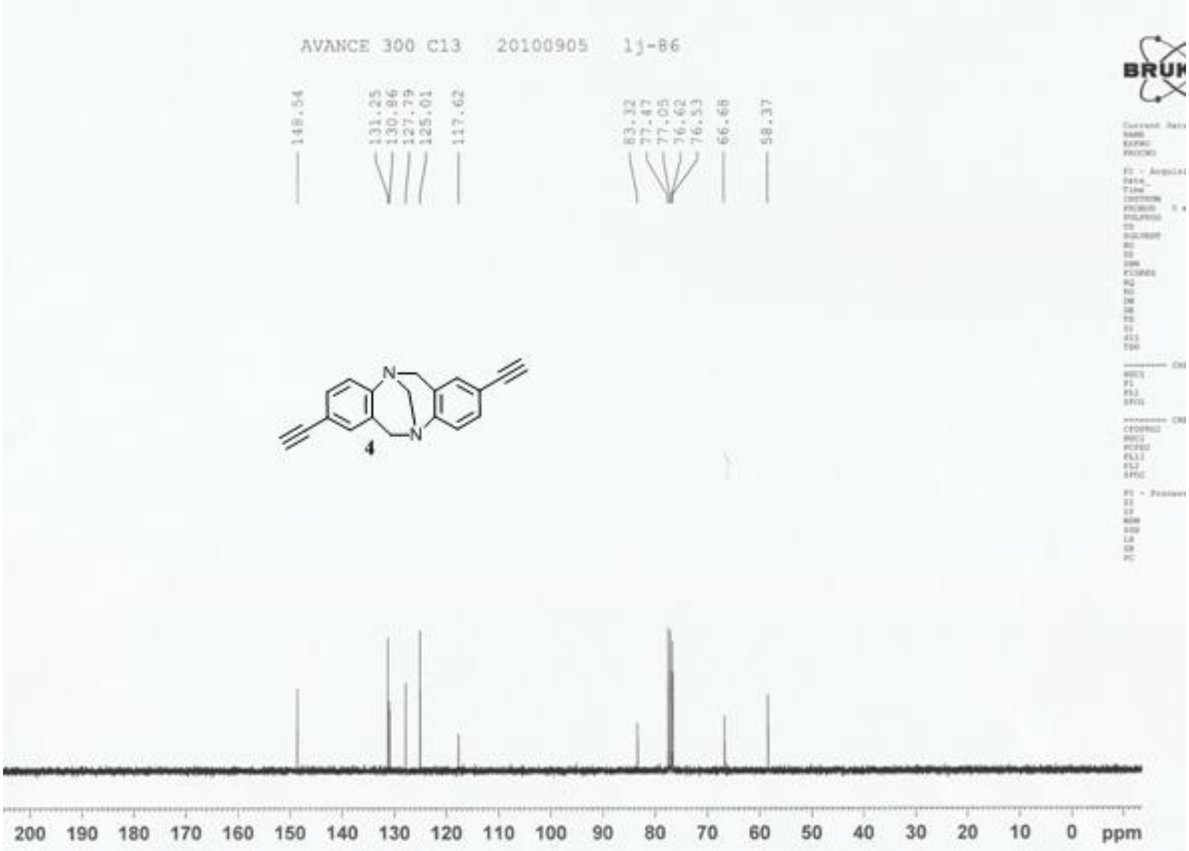

Figure 3(b). ^{13}C NMR spectrum of **4** (100MHz, $CDCl_3$)

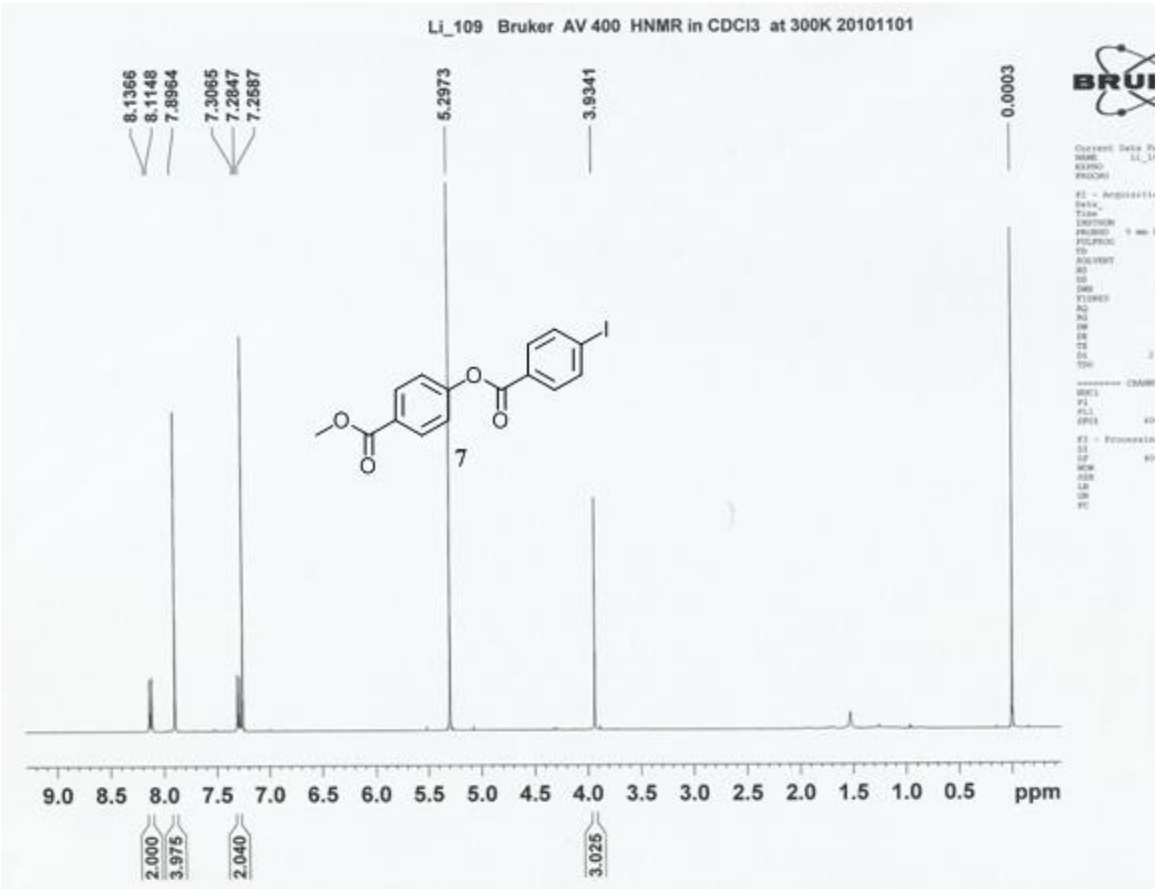

Figure 4(a). ^{1}H NMR spectrum of **7** (400MHz, $CDCl_3$)

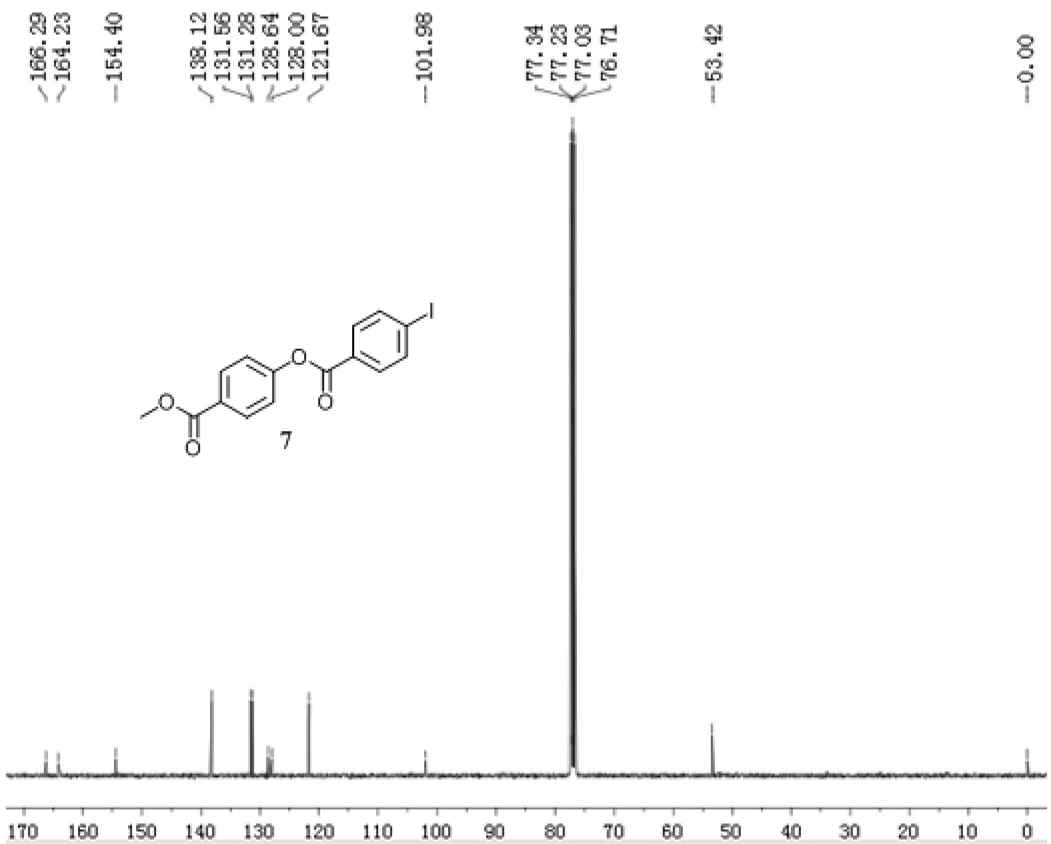

Figure 4(b). ^{13}C NMR spectrum of **7** (100MHz, $CDCl_3$)

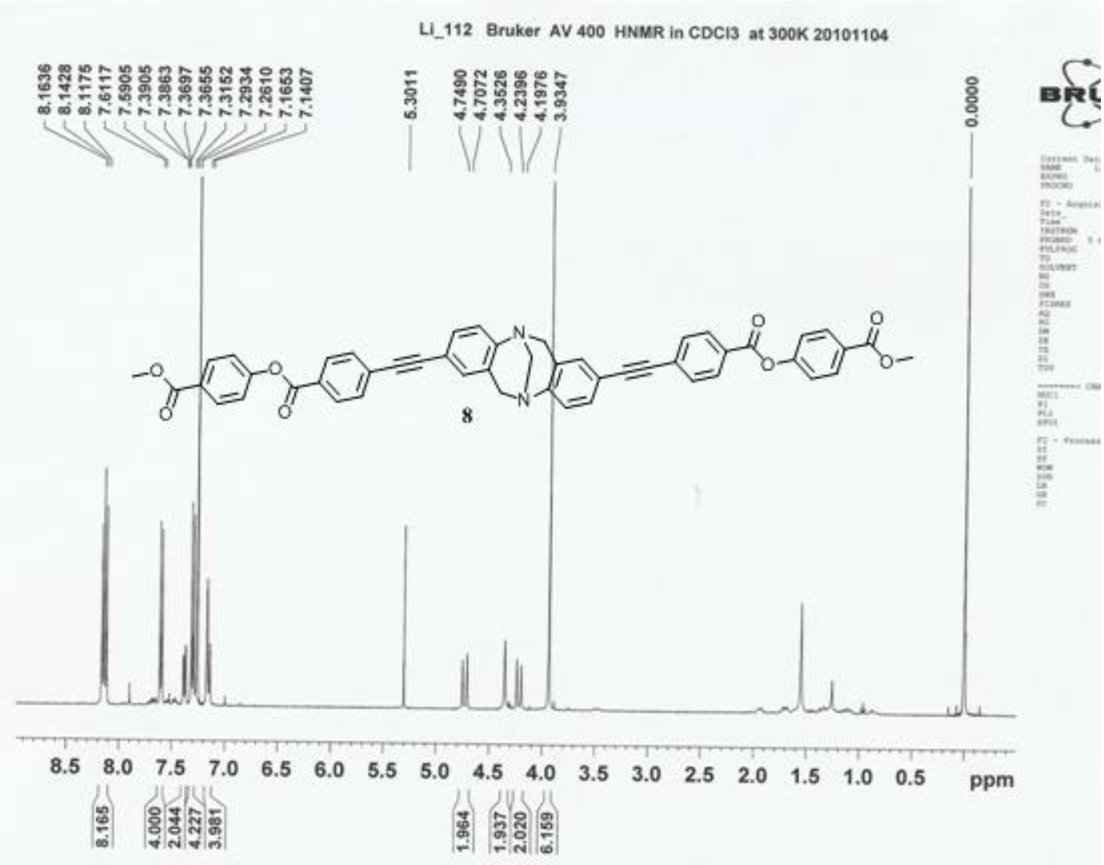

Figure 5(a). ^{1}H NMR spectrum of **8** (400MHz, $CDCl_3$)

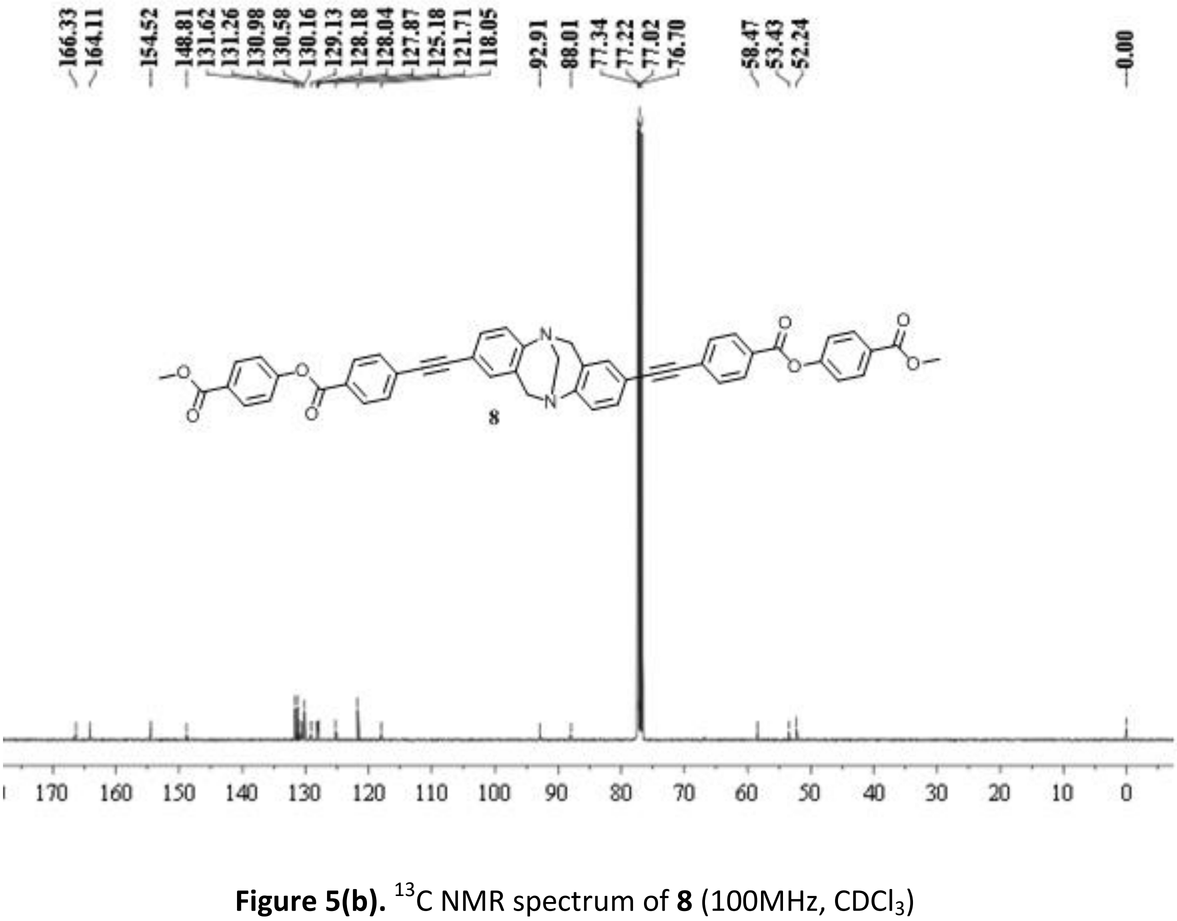

Figure 5(b). ^{13}C NMR spectrum of **8** (100MHz, $CDCl_3$)

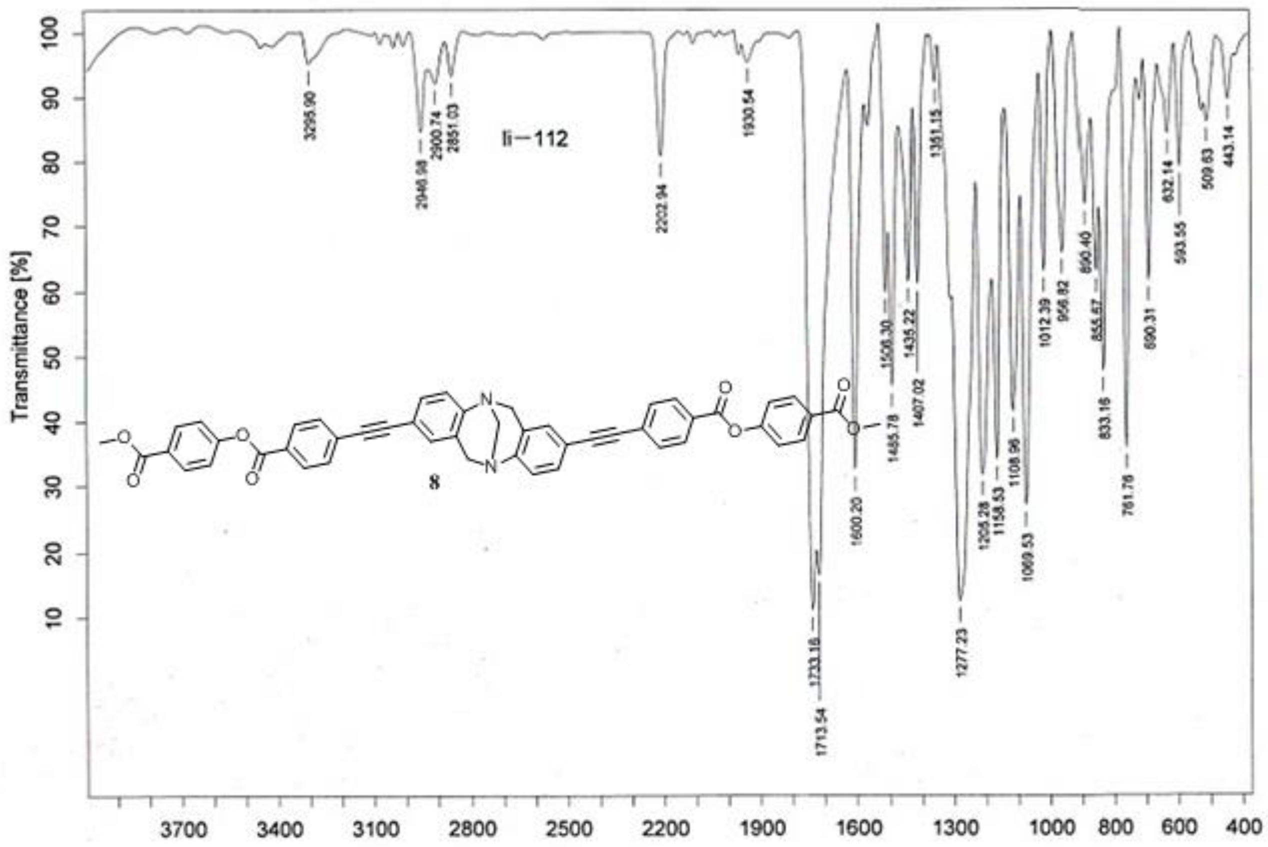

Figure 5(c). FT-IR spectrum of **8** (KBr).

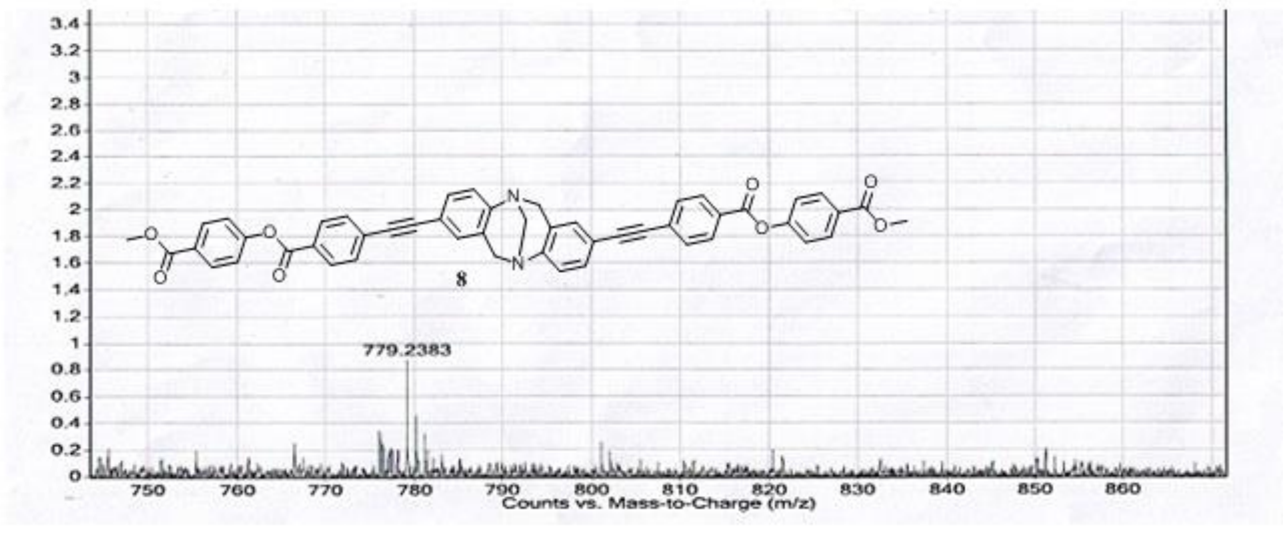

Figure 5(d). MS of 8. (ESI)
